# Classification of Types of Stuttering Symptoms Based on Brain Activity

**DOI:** 10.1371/journal.pone.0039747

**Published:** 2012-06-25

**Authors:** Jing Jiang, Chunming Lu, Danling Peng, Chaozhe Zhu, Peter Howell

**Affiliations:** 1 State Key Laboratory of Cognitive Neuroscience and Learning, Beijing Normal University, Beijing, People's Republic of China; 2 Division of Psychology and Language Sciences, University College London, London, United Kingdom; French National Centre for Scientific Research, France

## Abstract

Among the non-fluencies seen in speech, some are more typical (MT) of stuttering speakers, whereas others are less typical (LT) and are common to both stuttering and fluent speakers. No neuroimaging work has evaluated the neural basis for grouping these symptom types. Another long-debated issue is which type (LT, MT) whole-word repetitions (WWR) should be placed in. In this study, a sentence completion task was performed by twenty stuttering patients who were scanned using an event-related design. This task elicited stuttering in these patients. Each stuttered trial from each patient was sorted into the MT or LT types with WWR put aside. Pattern classification was employed to train a patient-specific single trial model to automatically classify each trial as MT or LT using the corresponding fMRI data. This model was then validated by using test data that were independent of the training data. In a subsequent analysis, the classification model, just established, was used to determine which type the WWR should be placed in. The results showed that the LT and the MT could be separated with high accuracy based on their brain activity. The brain regions that made most contribution to the separation of the types were: the left inferior frontal cortex and bilateral precuneus, both of which showed higher activity in the MT than in the LT; and the left putamen and right cerebellum which showed the opposite activity pattern. The results also showed that the brain activity for WWR was more similar to that of the LT and fluent speech than to that of the MT. These findings provide a neurological basis for separating the MT and the LT types, and support the widely-used MT/LT symptom grouping scheme. In addition, WWR play a similar role as the LT, and thus should be placed in the LT type.

## Introduction

Whilst most children acquire speech effortlessly, around 5% of children start to stutter usually between 2 and 6 years of age for reasons that are not entirely understood. Only a minority of the children who start to stutter (about 20%) continue into adulthood, and the problem is then referred to as persistent developmental stuttering.

Johnson and associates [1] proposed that the following symptoms were commonly observed in stuttered speech: 1) Incomplete phrases; 2) Revisions; 3) Interjections; 4) Phrase repetitions; 5) Whole-word repetitions (WWR); 6) Part-word repetitions; 7) Prolongations; and 8) Broken words. Languages other than English have found this taxonomy of symptom types useful in assessing stuttering. Thus, symptom-incidence has been used to assess stuttering in languages as diverse as Japanese [2] and Mandarin [3], [4]. Johnson and associates were aware that none of the listed symptoms is exclusive to people who stutter. Consequently, subsequent authors have attempted to identify which symptoms from this list are the most salient characteristics of stuttering by specifying which are more, and which are less, typical of stuttering (MT and LT respectively) [5]–[11].

Comparison of some of the best-known grouping schemes show that there is substantial agreement about which symptoms should appear in MT and LT. Conture's [12] scheme considers symptoms that happen within words (Johnson and associates' categories 5–8) are a sign of stuttering (MT). Yairi and Ambrose's [8] scheme places these same symptoms into the MT (which Yairi and Ambrose term stuttering-like disfluencies). Wingate's [11], [13] scheme divides the MT symptoms (types 6–8) from hesitation-type LT symptoms (types 1–5). Thus, all three schemes place symptoms 1–4 in the LT, and symptoms 6–8 in the MT [8], [11], [12], [14], [15].

Despite the fact that neural imaging research on stuttering has been conducted for more than a decade, there has been no neuroimaging evidence that supports such a symptom grouping scheme. The neuroimaging research shows that patients with stuttering have functional anomalies in the right frontal operculum/anterior insula, temporal areas, basal ganglia, and cerebellum [3], [16]–[23]. Patients who stutter also show altered connectivity between the basal ganglia/cerebellum and the cortical areas, and among different cortical areas [3], [4], [24], [25]. Studies that have examined brain structural anomalies have identified several anomalous brain regions, especially the left inferior frontal cortex (IFC), in persistent devleopmental stuttering [26]–[30]. However, it is not clear whether and how these neural anomalies are related to different stuttering symptoms. The current study aimed to examine whether different types of stuttering symptoms can be classified based on brain activity. This study was intended to provide neuroimaging evidence about the symptom grouping schemes descibed above.

Another question about stuttering symptoms that divides opinion is whether WWR are a core feature of the disorder and should be designated as instances of the MT type. Looking at clinical work first, the World Health Organization lists WWR as an MT whereas the most frequently used instrument for assessing stuttering omits them [5] and the Royal College of Speech Language Therapists in the UK does not mention WWR as MT features. Whether or not to include WWR in the MT has important practical implications as it affects diagnosis, outcome-assessment etc, of stuttering. Consequently, some of the authors mentioned have tried to qualify the circumstances in which WWR are, and are not, considered as part of the MT. Thus, Conture [12] voiced his ambivalence about the status of WWR, Yairi and Ambrose [8] have introduced a revised version of their stuttering-like disfluencies scheme which gives more weight to symptoms 6–8 than WWR, and Riley [5] mentioned that WWR may be considered stutters in exceptional circumstances.

There is also empirical evidence that supports the position that WWR have a different role to the remaining MT symptoms. For instance, WWR are not influenced by variables that affect the other symptoms in MT [31]. Further evidence suggests that WWR may have a specific role in promoting recovery from stuttering. There is little opportunity for speakers to produce WWR in Japanese because of the structure of the language [2]. Consequently, if WWR have a role in recovery, then recovery rates should be lower in that language than in speakers of Western languages as Ujihira [2] reported. Two further findings are potentially related to the role of WWR in recovery. First, those English-speaking children who show a preponderance of WWR are more likely to recover [32]; Second, the risk of persisting in stuttering is accurately predicted from Riley's [5] severity instrument, that excludes WWR in its assessment of stuttering [33]. Thus, the question of whether WWR should be placed in MT or LT was examined in the current study.

In sum, two questions were addressed: (1) whether neural processing is different for the MT and LT types (WWR excluded); and (2) whether WWR belong to the MT or LT type. Addressing these questions using fMRI data is important as the answers provided have a bearing on how stuttering is diagnosed and how to examine patterns of change in stuttering that occur spontaneously (natural recovery) or as a result of treatment. Any answers provided may also suggest hypotheses about what leads stuttering to start and indicate stuttering symptoms are related to other speech production disorders.

Pattern classification has been widely employed with fMRI data to predict unknown cognitive states (e.g., type of symptoms) or patient cases [34]–[40]. In the present study, this approach was used to address the above two questions. Specifically, during the experiment a sentence completion task was performed by the participants while they were scanned. This task elicited stuttering in adults with persistent developmental stuttering. Each stuttered trial from each patient was sorted into two types (MT, LT) with WWR put to one side. A support vector machine (SVM), which is a widely-used pattern classification method, was employed to train a patient-specific single trial classification model to automatically classify each trial as MT or LT using the corresponding fMRI data as the classification feature. This classification model was then validated by using test data that were independent of the training data. High classification accuracy would indicate that different neural systems underlie MT and LT and thus confirm the grouping schemes. In a subsequent analysis, the established classification model was used to determine which type the WWR should be placed in.

## Materials and Methods

### Ethics Statement

The study was approved by the ethics committee of the State Key Laboratory of Cognitive Neuroscience and Learning, Beijing Normal University. Written informed consent was obtained from each patient.

### Participants

Twenty male native Mandarin-speaking patients who stuttered were recruited. They were all right handed (mean score of 80±21) [41] and did not have a history of psychiatric or neurological disorders other than their stutter. All had started to stutter before teenage. Their mean age was 26.8±6.5 years. The ages at onset of stuttering and ages at the time of the test confirmed that these were adults with persistent developmental stuttering. None had been involved in a treatment program for at least six months prior to participation in the experiment. Stuttering at the time of the test was confirmed using a Mandarin translation of the Stuttering Severity Instrument Version III (SSI-3) [5]. This employed video recordings of a sample of spontaneous speech and a read text (both of which were at least 300 syllables long). Stuttering severity varied from mild to very severe. A summary of the information about the patients is given in Table 1, which also includes Overall Assessment of the Speakers' Experience of Stuttering (OASES) scores [42]. The latter assessment evaluates the experience of the stuttering disorder from the perspective of individuals who stutter.

**Table 1:** Demographic, diagnostic and symptom information for each patient.

Number	Age	Handedness	%SS	SSI-3	OASES	Stuttering Symptoms					
						PAUSE	MR	WWR	PWR	PRO	BREAK
1	21	54	11	25	53	18	4	2	9	1	1
2	34	40	11	32	39	9	1	0	13	4	0
3	18	82	13	28	66	17	7	3	6	2	0
4	36	60	9	24	48	12	1	2	8	3	0
5	23	80	11	25	49	16	0	0	5	0	0
6	29	100	10	22	57	18	4	3	4	2	0
7	23	100	11	28	74	19	1	1	13	13	1
8	31	100	13	38	56	12	5	2	7	4	4
9	37	100	24	24	71	9	2	1	8	6	1
10	17	80	11	22	57	8	5	2	8	3	0
11	24	100	7	32	51	12	2	1	5	6	1
12	38	100	13	27	63	10	3	4	22	10	0
13	22	60	10	28	50	18	1	3	9	3	0
14	25	100	13	32	52	10	0	0	38	1	0
15	24	100	11	25	48	15	3	0	3	2	0
16	26	68	13	29	46	12	7	2	4	1	1
17	36	100	7	18	39	32	5	1	6	5	0
18	29	64	11	29	37	23	0	0	14	4	0
19	21	62	16	32	67	15	10	4	16	0	0
20	22	50	16	43	67	7	1	0	66	10	0
Mean	26.8	80	12.05	28.15	54.5	15	3	2	13	4	1
SD	6.54	20.91	3.66	5.72	10.76	5.93	2.75	1.36	14.79	3.52	0.94

Scores in each of the symptom subtype columns are number of trials of the subtypes indicated in the sentence completion task.

Note: %SS, percent of stuttered syllable; SSI-3, Stuttering Severity Instrument Version III; OASES, Overall Assessment of the Speakers' Experience of Stuttering. PAUSES  =  pauses between characters, and prolongation of the rhythm part of the character; MR  =  multiple-character repetition; WWR  =  whole-character repetition (including the tone). This is equivalent to monosyllabic whole-word repetitions in English; PWR  =  repetition of onset consonants; PRO  =  prolongations of onset sounds. BREAK  =  word breaks.

### Experimental tasks and materials

Ninety simple sentences with the same grammatical structure were generated that varied in length between 8 and 10 Mandarin characters (each character represents a syllable). For each sentence, only the stem (subject and predicate, 5–6 characters in length) was retained to provide probes for the sentence-completion task. Twenty fluent participants, who were not involved in the experiment, assessed how appropriate each stem was for sentence completion and the familiarity of the stems. Five-point scales were used for both these judgments (high scores indicated that the stems were appropriate and familiar), and mean scores were 4.22±0.94 and 3.93±1.03, respectively. The sentence stems were then read and recorded by a female Mandarin speaker (the average duration of stems was 1700 msec).

An event-related design was employed. A third of the trials were null trials, i.e., involved no sentence stimuli and no response was required. Design parameters that optimized number and timing of acquired data points and timing of the events that were targeted, based on pilot data, were then obtained using the Optseq2 toolbox (http://surfer.nmr.mgh.harvard.edu/optseq/). The 90 sentence stems plus 30 null trials were split into two scanning runs. The two runs were counterbalanced across the patients. Two null trials were added at the beginning of each run, which ensured the first two trials that were dropped during analysis were not task trials.

**Figure 1:**
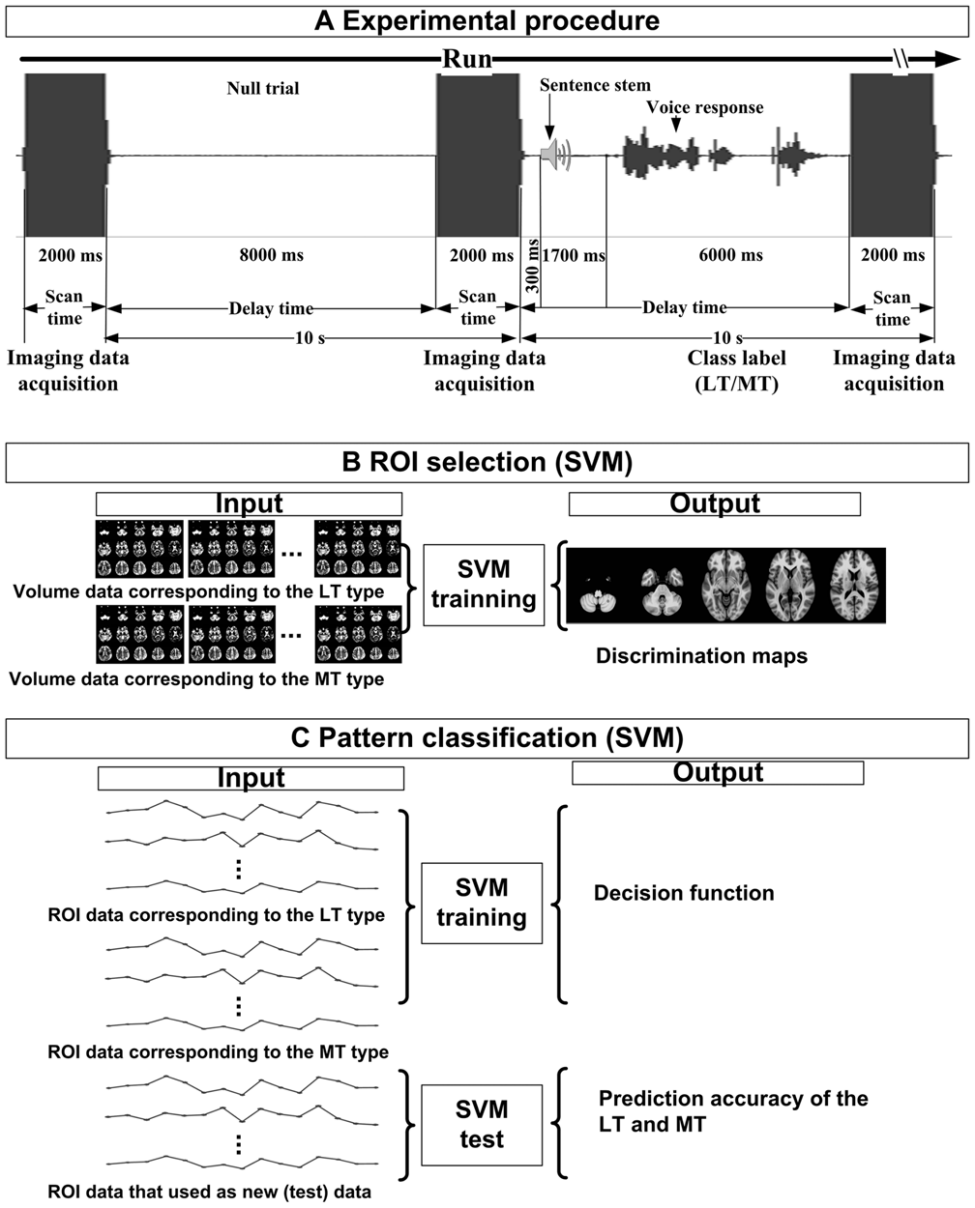
Illustration of the experimental analysis procedure. (A) Experimental procedure of the sparse sampling technique. The sequence for two trials is illustrated (one null trial and one task trial). For both types of trial, there was an 8 sec delay (silent interval) and 2 sec imaging data acquisition. During the silent interval on a null trial, no sentence stimulus, nor verbal response was required. During the silent interval of a task trial, after a 300 msec pause, the sentence stem was aurally presented and lasted for about 1700 msec. The remaining 6 sec were left for the patients to complete the sentence aloud. Note that both the auditory stimulus and the verbal response fall within the silent interval before imaging data acquisition. The speech waveform represents the overt response of patients, which were recorded by an fMRI-compatible microphone. (B) ROI selection using the SVM method. For each patient, the input data were fMRI volume data corresponding to a trial assigned to MT or LT., An output discrimination map was produced by SVM training. T-tests on the discrimination maps across patients identified ROIs. (C) Pattern classification based on the selected data within each ROI. The performance of the MT-LT classification was evaluated using the leave-one-trial-out cross validation test.

During the experiment, patients fixated on a spot at the center of the experiment-control screen. During null trials they continued fixation without any movements. During task trials, after a pause of 300 msec, a sentence stem was played to the patient via MRI-compatible headphones. The headphones delivered high quality sounds and attenuated background noise. When presentation of the sentence stem stopped, the patient was required to complete the sentence as quickly as possible. Patients were allowed a maximum of 6 sec to complete the sentence and responses were recorded. Each trial during the task was then sorted into different types in order to obtain class labels (see below). To ensure that patients completed the task within the 6 sec after the probe, an indication of time remaining was given on the screen. After this period, the fixation sign appeared on the screen for 2 sec. The patient was scanned during this phase to capture the neural response to the sentence completion task. The captured neural responses were used as the classification features for pattern classification. The experimental procedure is illustrated in Figure 1A.

### Imaging data acquisition

Imaging data were acquired on a Siemens TRIO 3T MR scanner. Patients lay supine within the scanner, their heads secured with foam padding. Structural images were obtained first from each patient. A high resolution T1-weighted MP-RAGE sequence was used: time repetition (TR)  = 2,530 msec; time echo (TE)  = 3.30 msec; flip angle  = 7°; slice thickness  = 1.3 mm; in-plane resolution  = 1.3×1.0 mm^2^; 128 interleaved sagittal slices. Then functional data were collected using a sparse sampling technique based on the BOLD response (Hall et al., 1999; Watkins et al., 2008). The T2-weighted axial gradient recalled echo planar images (EPI) were acquired with the following parameters: TR = 10,000 msec (delay  = 8,000 msec); TE = 30 msec; flip angle  = 90°; field of view  = 200 mm; matrix  = 64×64; slice thickness  = 4.8 mm; in-plane resolution  = 3.1×3.1 mm^2^; 33 interleaved axial slices.

### Speech data assortment

Each task trial was assigned to one of four types based on the symptoms contained [15]. This was done by two senior researchers who are native Mandarin listeners. One researcher had more than 9 years' experience judging speech, whereas the other had 2 years' experience. The types were: *Type one*, fluent: These were task trials where there was no stuttering. The following two symptoms were considered fluent; First, planning pauses that occurred between the sentence stem that was played to the patient and the part that was added by the patient; Second, prolongations that occurred on the last character of the sentence stem were regarded as being due to pre-pausal lengthening (a feature of fluent speech); *Type two*, LT: Task trials that involved the following symptoms were classed as LT: Multiple-character repetition (including the tone) (MR), which are sometimes phrase repetitions and sometimes multi-character word repetitions; Prolongation of the rhyme part of non-final characters, which correspond to fluent elongation of rhyme (equivalent to drawling on English words), and pauses between characters (PAUSE); *Type three,* MT: The symptoms for this type had the general characteristic that they involved interruption of the phones within syllable characters. Specific symptoms were: Prolongations of onset sounds (PRO); Repetition of onset consonants (these are predominantly single consonants in Mandarin, and they correspond to part-word repetitions in English) (PWR); Breaks between the phones within a character (BREAK); *Type four*, WWR: Monosyllabic WWR were singled out to test which type (MT or LT) they should be grouped into.

As reported for English, only a few task trials showed both the MT and LT (at any position in the sentence). These were excluded from all subsequent analysis. Fluent task trials were also excluded because the focus of the study was on the neural differences between stuttering symptoms. Thus, only pure type two and pure type three were used during the analyses to establish the model for classifying MT and LT. The intra-class reliability on classifying stuttering symptoms from individual patients ranged from 0.85 to 0.99, which indicated a high-level reliability. The number of stutters each patient produced in the sentence-completion task broken into different subtypes are summarized in Table 1. As Table 1 shows, the number of the MT was about equal to that of the LT which ensured the analysis had equivalent power for separating these two types of stuttering symptoms.

### Imaging data analysis

During the imaging data analysis, the first scanning run was used as a localizer run to select region of interest (ROI). The selected ROIs were then used as pointers to select data from the second scanning run for use in pattern classification (see below).

#### Pre-processing

The first two volumes of the functional images were discarded prior to data analysis to allow the magnetic field to stabilize. During pre-processing, slice-time correction, image registration, motion correction, and spatial smoothing (full width half maximum  = 6 mm) were performed using Analysis of Functional NeuroImages (AFNI, http://afni.nimh.nih.gov/afni) [43], [44]. The pre-processed time course of each voxel was then converted into percent signal change. Finally, individual images were normalized to Montreal Neurological Institute space.

#### ROI selection

When applying pattern classification to fMRI data, the number of voxels that convey the discriminative information is small compared to the total number of measured voxels. This leads to an overfitting problem that degrades performance [45]–[47]. To reduce the data dimensionality, previous studies have employed various methods to select voxels (i.e., brain regions) [34], [39]. Recently multivariate methods such as SVM have been shown to provide superior performance to the univariate voxel selection ones and pattern recognition with no voxel selection [35]. Here, SVM was used to select ROIs based on the first scanning run data (see Figure 1B). The ROIs were then used on the second scanning run to establish the classification model.

Specifically, a linear kernel SVM algorithm was used to analyse the first scanning run data, and used to obtain a discriminating map for each patient (3dsvm program in AFNI) [38]. The absolute magnitude of each voxel within the discriminating map determined its importance in classifying MT and LT [39]. In order to obtain a consistent map across patients, a random-effect one-sample two-tailed *t*-test was then conducted. A threshold of *P*<0.05 was used to select ROIs (corrected by Monte Carlo simulation with a cluster size threshold >327 mm^3^, individual voxel *P*<0.01) [48], [49].

#### Classification model training and validation

The averaged BOLD signals within each ROI were calculated from the second scanning run and fed into the SVM as the classification feature. The details about the SVM approach are available elsewhere [36], [37], [39], [40], and are illustrated in Figure 1C. The trained classification model was validated using the leave-one-trial-out cross validation test [50]. To quantify the performance of the predicted classifications, sensitivity, specificity, and generalization rate of the prediction were defined using observed and predicted results (see foot of Table 2 for definitions). To identify brain regions that contributed significantly to the discrimination of the classification model, the weighted coefficients of each ROI, which represented its importance for discrimination, was calculated from the classification model for each patient. Then, one-sample two-tailed *t*-tests were conducted across patients on these ROIs.

**Table 2:** Parameters to quantify the performance of the classifier.

Observed	Predicted		
	1	2	Percent Correct
1	TP	FN	Sensitivity
2	FP	TN	Specificity
Overall Percentage			Generalization Rate

Note: 1 and 2 represents two conditions. TP (true positive) is the number of LT symptoms correctly predicted; TN (true negative) is the number of MT symptoms correctly predicted; FP (false positive) is the number of MT symptoms classified as LT symptoms; FN (false negative) is the number of LT symptoms classified as MT symptoms. Sensitivity indicates the proportion of LT symptoms correctly predicted, the specificity indicates the proportion of MT symptoms correctly predicted, and generalization rate is the overall proportion of samples correctly predicted. These were calculated as follows: Specificity  =  TN/(TN + FP); Sensitivity  =  TP/(TP + FN); Generalization Rate  =  (TP + TN)/(TP + FN + TN + FP).

#### Prediction of the class of WWR

As stated in the introduction, it is not clear which type, i.e., MT or LT, WWR should be placed in. Thus, after the classification model had been established, it was used to determine which type WWR trials belonged to. Specifically, fMRI data of WWR trials were fed into the classification model as unclassified cases, and the outcome was the predicted class label

## Results

### Speech data assortment

The average numbers of the trials in LT and MT across the patients were 18 (*S.D* = 6.81) and 18 (*S.D* = 16.38), respectively. There was no significant difference between the numbers in LT and MT (Wilcoxon Signed Ranks Test, *Z* = 1.047, *P* = 0.295). The average number of trials in WWR was 2 (*S.D* = 1.36). The detailed information for the subtypes of LT and MT are provided in Table 1. The numbers of each subtypes of stuttering symptoms in the first and second run are also shown in Table 1. It should be noted that the number of LT did not differ significantly from the number of MT in both the first run (*Z* = −1.409, *P* = 0.159) and the second run (*Z* = −0.2, *P* = 0.984). Moreover, the number of LT and MT did not differ significantly between the first and the second run (LT, *Z* = −1.817, *P* = 0.069; MT, *Z* = −0.415, *P* = 0.678).

### ROIs

**Figure 2:**
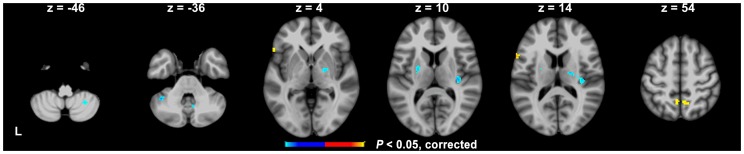
The ROIs for pattern classification identified on the basis of the first scanning run data. The yellow and blue blobs indicate brain areas that contribute significantly to the classification of LT and MT, respectively (*P*<0.05, corrected). L is left, B is bilateral.

Statistical tests on the activation map found that the left IFC (BA44/45) and bilateral precuneus (one cluster covering both hemisphere, BA7) showed significantly positive values (see yellow blobs in Figure 2). This suggests that these brain regions had higher brain activity for MT than for LT. The bilateral basal ganglia (including the bilateral putamen and the right lateral global pallidus, LGP) showed significantly negative values (see blue blobs in Figure 2). By lowering the cluster size threshold, the right cerebellum VIII also showed a negative value (*P*<0.001, uncorrected). This suggests that these brain regions had higher brain activity for LT than for MT. The statistics are summarized in Table 3. These brain regions were selected as ROIs.

**Table 3:** Brain regions that were selected for pattern classification.

Brain region	Position			*t*-value	cluster volume (mm^3^)
	x	y	z		
**Higher value in the MT than in the LT**					
Left Inferior Frontal Cortex (BA44/45)	−59	14	14	3.976	432
Precuneus (BA7)	−4	−54	54	3.101	1008
**Lower value in the MT than in the LT**					
Left Putamen	−24	−5	10	−3.484	432
Right Putamen	30	−16	15	−3.908	1656
Right Lateral Globus Pallidus	24	−4	4	−3.298	352
Left Cerebellum	−40	−48	−36	−4.151	384
Right Cerebellum (VIII)*	30	−58	−46	−3.25	96

Note: * the right cerebellum (VIII) did not survive the cluster size threshold.

### Classification model performance in classifying MT and LT

**Figure 3:**
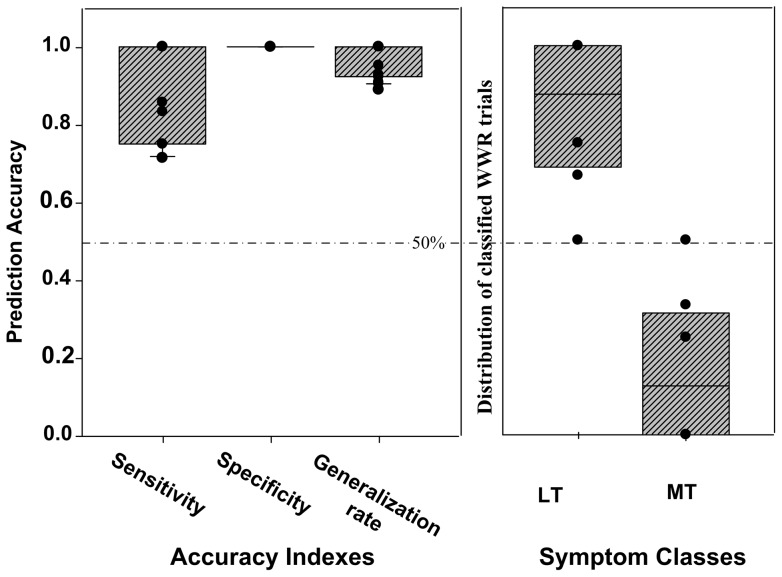
Classification accuracy of the classification model. Left part: sensitivity, specificity, and generalization rate of the classification model; right part: prediction accuracy of WWR for LT and MT, respectively. The black dots indicate the distribution of each patient's data, and the bars indicate the 5% and 95% confidence interval.

**Figure 4:**
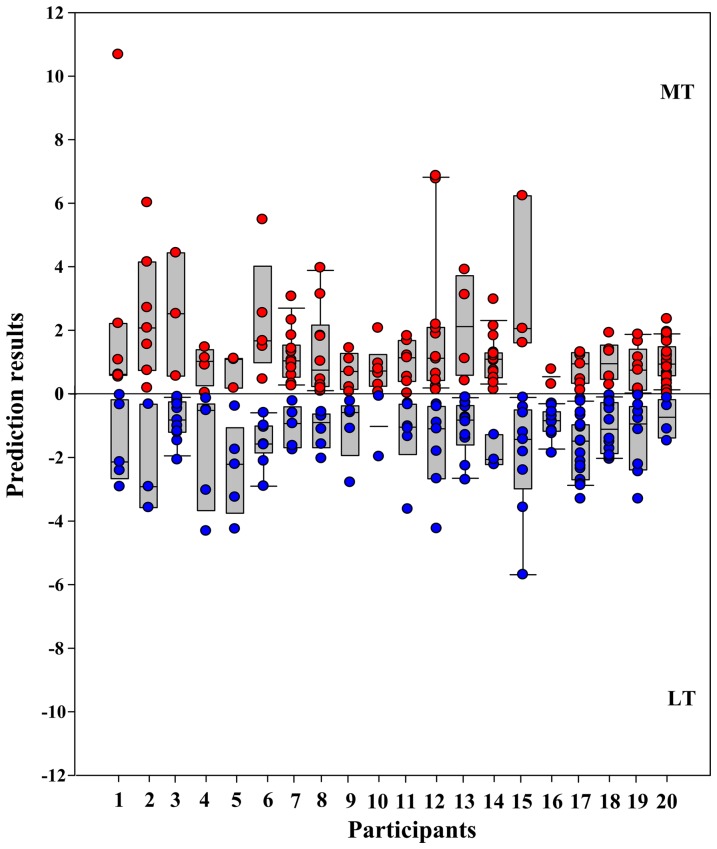
Classification results for each patient. The x axis represents individual patients and the y axis represents the prediction results. The more negative (lower part, blue dots) or positive (upper part, red dots) the value on the y axis is, the better the classification performance is, for each individual patient. Each bar indicates the mean value and the 5% and 95% confidence interval of the correctly predicted results. Each dot indicates a single prediction during leave-one-out cross-validation.

Based on the second scanning run's fMRI data, results of the pattern classification showed that the average sensitivity, specificity, and generalization rates for classifying the types of stuttering symptoms were 0.91 (*S.D* = 0.12), 1 (*S.D* = 0) and 0.97 (*S.D.* = 0.04) (see Figure 3, left part), respectively. Statistical tests showed that sensitivity and generalization rate were significantly higher than chance level (.5) (Sensitivity: *t* = 14.727, *P*<0.001; Generalization rate: *t* = 47.048, *P*<0.001). The specificity was 1 for all patients. The within-patient variability across the LT and MT trials is shown in Figure 4. The performance statistics supported the conclusion that LT and MT were associated with different brain activity patterns, and thus support the distinction that they are different types of stuttering symptoms.

**Figure 5:**
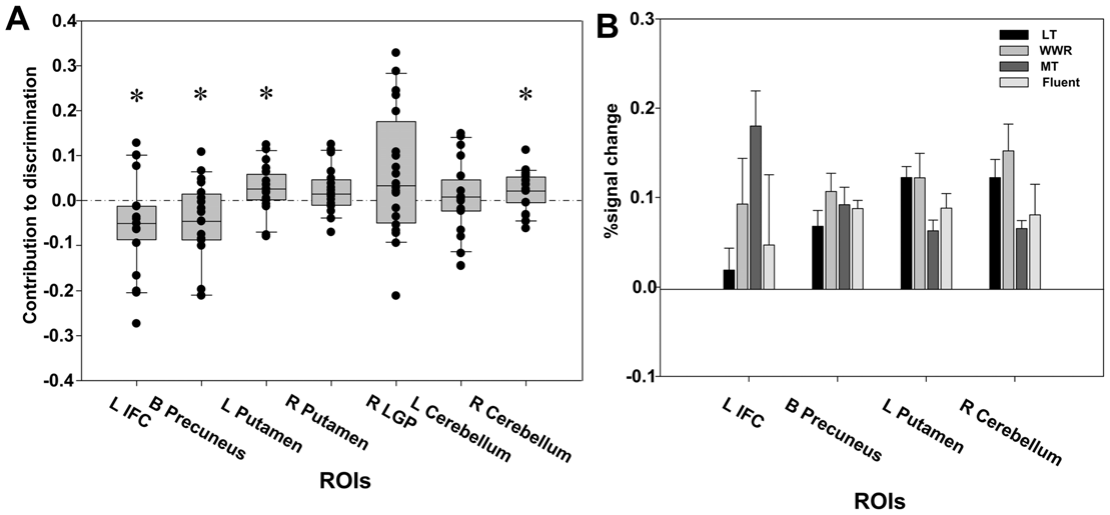
Role of each ROI in each type of stuttering symptom. (A) The contribution of each ROI to the discrimination performance. (B) Averaged pre-processed BOLD signal in LT, WWR, MT, and fluent speech, respectively. The error bars indicate standard errors. Stars indicate significance at *P*<0.05 level.

Statistical test on ROI's weighted coefficient showed significant contribution to the classification of LT and MT in the left IFC (*t* = −2.188, *P* = 0.041), bilateral precuneus (*t* = −2.346, *P* = 0.03), left putamen (*t* = 2.188, *P* = 0.041), and right cerebellum (*t* = 2.214, *P* = 0.039), whereas the contributions in the right putamen (*t* = −2.018, *P* = 0.058), right LGP (*t* = 1.873, *P* = 0.077), and left cerebellum (*t* = 0.439, *P* = 0.666) did not reach significance (see Figure 5A). The sign direction (i.e., negative or positive value) of the ROIs indicated that the left IFC and bilateral precuneus had higher brain activity in MT than in LT, whereas the left putamen and right cerebellum had the reverse pattern. These results confirmed that the left IFC and precuneus were more closely associated with MT, whereas the left putamen and right cerebellum were more closely associated with LT.

To further confirm the above results, the pre-processed BOLD signal was averaged across the MT, LT, and fluent speech trials (*Type four*), respectively, based on the second scanning run data (see Figure 5B). Statistics showed that the left IFC showed significantly higher neural response in MT than in LT (*t* = 3.452, *P* = 0.003). For the left putamen and right cerebellum, the results also confirmed the above results by showing significant differences in neural response between MT and LT (left putamen: *t* = −3.822, *P* = 0.001; right cerebellum: *t* = −2.194, *P* = 0.041). No significant effect was found in the precuneus (*t* = 0.947, *P* = 0.355). These results confirmed that MT and LT differed in the associated brain activity patterns, and thus represented different types of stuttering symptoms.

Additional findings were that there was a trend that, in the left IFC, the neural response in fluent speech was more similar to that in LT (*t* = −0.368, *P* = 0.717) than to that of MT (*t* = 1.476, *P* = 0.156), whereas in the left putamen and right cerebellum, the neural response in the fluent speech was more similar to the activity in MT (left putamen: *t* = 1.25, *P* = 0.227; right cerebellum: *t* = 0.41, *P* = 0.686) than that in LT (left putamen: *t* = 1.606, *P* = 0.125; right cerebellum: *t* = 1.414, *P* = 0.174). These findings further support the view that MT and LT were associated with different brain activity patterns.

### Prediction of the type of symptom WWR belong to

The average accuracy of classifying WWR trials as of the LT type was 0.83 (*S.D* = 0.19), whereas that of classifying WWR as of the MT type was 0.17 (*S.D* = 0.19) (see Figure 3, right part). The accuracy of classifying WWR as of the LT type was significantly higher than chance level (0.5) (*t* = 4.872, *P* = 0.002), whereas classification as of the MT type was not (*t* = −4.872, *P* = 0.002). Based on these findings, it can be concluded that the brain activity of WWR suggest that it is a member of LT, not MT.

Comparison of pre-processed BOLD signal responses for WWR in regions associated with LT showed no significant difference between WWR and LT but there were difference between WWR and MT (see Figure 5B). Specifically, the right cerebellum showed a significant difference in neural response between MT and WWR (*t* = −2.843, *P* = 0.01), but not between LT and WWR (*t* = −0.781, *P* = 0.444). Similarly, in the left putamen, the difference in neural response between MT and WWR approached significance (*t* = −1.961, *P* = 0.065), but was not significant between LT and WWR (*t* = 0.007, *P* = 0.994). In contrast, the left IFC, which was associated with MT, showed no significant difference in neural response between LT and WWR (*t* = −1.247, *P* = 0.228) nor between MT and WWR (*t* = 1.233, *P* = 0.233). This suggested that 1) the brain regions that were associated with LT were more sensitive in classifying WWR and 2) WWR should be placed in LT, not MT. Finally, BOLD responses in these regions on fluent speech trials were examined: The differences with WWR were not significant for the right cerebellum (*t* = −1.717, *P* = 0.102), the left putamen (*t* = −0.977, *P* = 0.341), and the left IFC (*t* = −0.466, *P* = 0.646). Overall, these findings further support the view that WWR is more similar to LT and fluent speech than to MT with regards to the brain activity patterns.

## Discussion

The present study examined grouping schemes for stuttering symptoms and the type of WWR. The results showed that different brain activity patterns were associated with MT and LT: while the left IFC and bilateral precuneus showed higher brain activity in MT than in LT, the left putamen and right cerebellum VIII showed the reverse pattern. Trials of MT-type and LT-type were correctly classified based on the brain activity in these regions. The present study also examined the assignment of WWR symptom into MT and LT which is an issue that has been debated for many years. The results showed that WWR should be placed into LT and that they were more similar to the fluent speech than to stuttered speech (i.e., MT). These results are discussed in detail below.

### The grouping schemes for stuttering symptoms

In contrast to LT, MT was closely associated with higher brain activity in the left IFC (see Figures 2 and 5). This finding is consistent with previous neurological evidence about stuttering. Structurally, the left IFC shows reduced grey matter volumes in stuttering patients [26], [30]. The fiber anomalies in stuttering patients were within late-myelinating associative and commissural fibers suggesting a myelogenesis-related neuro-developmental deficit in stuttering patients [27]. Stuttering patients also showed a reversed functional activation sequence and dysfunctional connections between the left IFC and the cortical and subcortical regions [3], [24], [51]. Other studies have reported functional and structural anomalies in brain areas that surround, or are connected with, the left IFC in stuttering patients [23], [29], [52]. However, it was not clear what roles the left IFC plays in stuttering, partly because this prior work did not distinguish between MT and LT. The present results showed that the anomaly in the left IFC was more closely associated with the core stuttering symptoms (i.e., MT) than symptoms that are common for both stuttering and fluent speakers (i.e., LT).

Another brain region that was more closely associated with MT than LT was the bilateral precuneus (see Figure 2 and Figure 5A). However, this brain region was not confirmed in the comparison of the pre-processed BOLD signal across MT and LT (see Figure 5B). This brain region has been reported to show lower activity in stuttering patients than in fluent controls during both speech and non-speech planning [20], but greater activity in stuttering patients than in fluent controls during imagined stuttering [53]. The bilateral precuneus also correlated negatively with stuttering severity after treatment [18]. Activity in this region was found to be involved in orthographic-phonological mapping [54] and auditory sound or word processing [55], [56] in control individuals. It is also involved in working memory, action, and visual spatial processing [57].

The results showed that LT was associated with higher activity in the classic motor regions of the brain, including the left putamen and right cerebellum VIII (see Figures 2 and 5). These are different brain regions to those identified when MT were produced. Several previous studies have reported significantly different neural activity in the basal ganglia in stuttering patients compared to controls, and where speech was disfluent or induced to be fluent in stuttering patients [4], [23], [58]–[60]. A significant correlation between activity in the basal ganglia and stuttering severity level has also been reported [18]. Similarly, the overactivation of the right cerebellum has been identified as one of the three neural signatures of stuttering [16] and was identified as specific to overt stuttered speech [25], [58], [59]. Furthermore, stuttering patients showed altered functional connectivity between the putamen/cerebellum and the cortical motor areas to controls [3], [4]. All these lines of evidence are consistent with the well documented role of the putamen and cerebellum in motor control [61], [62] and speech production [63]. Further evidence has shown that the basal ganglia play a key role in providing internal timing cues to the supplementary motor areas, whereas the cerebellum provides external timing cues to the premotor area, during motor control [64]–[66].

One possible explanation for the above findings is that MT may reflect a linguistic processing deficit. Theories such as CRH [67] and EXPLAN [68] concur with this view about what symptoms should be placed in MT. EXPLAN theory explicitly proposed that stutters which occurred on word fragments (MT) reflect phonological processing difficulty [68]. The proposition that MT is a direct response to a linguistic deficit is also supported by previous extensive neuroimaging evidence about non-stuttering people. Convergent evidence has shown that the degree of activation of the left IFC in normal speakers and damage to the left IFC in aphasic speakers were associated with performance on a speech production task [69]. This region is particularly associated with lexical selection [69], phonological processing [70], phonetic encoding [71], [72], and integration of this information [73]. However, the left IFC and bilateral precuneus are also involved in motor functions, such as motor sequence learning and action observation and imitation [74], [75]. Thus, it is possible that MT is associated with both linguistic processing and motor control deficits in stuttering.

A similar conclusion can be drawn about LT. On the one hand, the present results showed that when LT occurred, motor regions, especially those in the right cerebellum, were involved, whereas no areas in the temporal cortex were. This finding may support the important role of the right cerebellum in detecting and correcting problems during speech production. Another possible account of the findings is that the anomaly in the motor regions reflects a motor control deficit in people who stutter [76]. However, it is difficult to explain why such a deficit is more evident on the LT than on the MT. Meanwhile, recent studies have shown that both the putamen and the cerebellum were involved in linguistic processing [77]–[80]. Thus, it cannot definitely be concluded whether LT are associated with linguistic processing problems or motor control deficits in stuttering.

Thus, it is possible that deficits in both linguistic processing and motor control results in MT and LT. Nevertheless, MT and LT are associated with brain activity patterns in different regions. Based on the activity of these brain regions, LT was classified with relatively low-level accuracy compared to MT (t = −3.432, *P* = 0.003). These findings suggest that LT conforms to the definition that this type of stuttering contains *less typical* symptoms that are more difficult to classify than MT. They further indicate that MT and LT are probably associated with different behavioural characteristics: MT exhibited more linguistic characteristics, whereas LT exhibited more motor characteristics. This conclusion is consistent with two recent studies. One of them found that during both planning and execution processes, people who stutter showed widely distributed differences in brain activity relative to those of fluent controls [20]. The other study found that people who stutter differed from fluent controls in both linguistic planning and articulation processes [3]. An additional finding in the latter study was that two separate neural circuits were associated with each of the processes: the basal ganglia-IFC/primary motor area associated with planning and the cerebellum-primary motor area with articulation. Overall, these findings suggest that the different behavioural characteristics between LT and MT may be well explained by different brain regions/activities that are associated with each of them.

### Assignment of WWR to a symptom type

The classification results showed that the accuracy of classifying WWR as LT was significantly higher than chance, whereas that of classifying WWR as MT was not (see Figure 3, right part). This result indicated that WWR was closer to LT, rather than MT. The examination of the pre-processed BOLD signal further confirmed this conclusion (see Figure 5B).

s one exhibited more linguistic characteristic, whereas the other exhibited more motor characteristics. s, the MT and LT showed As stated in the introduction, WWR are a symptom that some authors designate as from MT, sometimes with reservations [8], [12] whereas others do not consider them to be from MT [11], [14], [15]. At present, all general speech production theories that apply to stuttering suggest a relationship between LT stuttering symptoms and motor control aspects of stuttering [15], [67], [81]. Moreover, the putamen and cerebellum have been shown to be a key brain region that provides timing cues for motor control [64]–[66]. Overall, WWR are close to LT, and are likely involved in motor control.

These results have clinical implications. As stated in the introduction, there has been controversy concerning which type WWR fall into. This issue is important because it affects the diagnosis of people who stutter and assessment of treatment outcome. It also affects the demographic estimates of early stuttering onset, recovery, and persistence. The present findings established that: WWR were similar to LT. Thus, WWR is likely to be a subtype of LT. This is not to say that WWR play no role in stuttering. For instance they play a role in promoting recovery [32] and are useful for diagnosis because they occur at higher rates in patients who stutter than in fluent speakers [82]. Furthermore, the number of WWR was small in the present study, which prevented us from further examining within-participant variability of brain activity corresponding to WWR or the neural network specifically associated with WWR. In future work, larger speech samples are required to further address these questions.

### Implications for future studies

The present study raises the possibility of augmenting behaviour-based stuttering diagnosis with brain activity-based automatic classification. The results reported showed that MT and LT could be classified at an accuracy level that was significantly above chance, based on the brain activity associated with stuttering symptoms. Furthermore, based on the established classification model, the type that WWR belonged to was determined to be LT. Overall these findings support an application of pattern classification in the field of the neurophysiology of stuttering, and diagnosis and treatment-assessment of stuttering.

In the present study, selection of the ROIs was based on group-level, rather than individual-level, data. Thus, these ROIs may not be able to explain the full variability of stuttering symptoms during classification. As shown by the results, the classification performance based on group-level data is good at 90% or above (see Figure 3, left part). This means that there is high-level consistency across individual patient's data (see Figure 4). However, if ROIs were defined based on each patient's data, the classification performance should be better than the current ones, and if so these would be more suitable for clinical diagnosis and treatment-assessment of stuttering than group-based ROI. This possibility will be explored in future work.

The present study employed a sparse sampling technique to acquire the neural responses in the sentence completion task. This technique ensured that scanner noise did not lead to a situation of speaking in noise, and avoided movement artifacts. Previous studies have shown that the hemodynamic response reaches its peak about 4–5 sec after presentation of the stimulus [83], [84]. Moreover, stuttering usually occurs in the early parts of utterances, and the sentence stem took an average of 1.7 sec to produce. Thus, the neural response to both the onset of the sentence stem and that of the part added by the participants were captured within the 2 sec scanning phase. Thus this technique may have special value for studying stuttering.

In sum, the current study used a neuroimaging method to examine the controversial issue of how to group different stuttering symptoms. The results provided neuroimaging evidence for the grouping scheme into MT and LT. It was shown that different brain activity patterns were associated with MT and LT, and each of these could be correctly classified based on the brain activity patterns. Further results showed that WWR were more similar to LT than they were to MT, with regards to the brain activity patterns. The present results have important theoretical and clinical implications.
